# Characterization of *Salmonella* Isolates from Various Geographical Regions of the Caucasus and Their Susceptibility to Bacteriophages

**DOI:** 10.3390/v12121418

**Published:** 2020-12-10

**Authors:** Khatuna Makalatia, Elene Kakabadze, Jeroen Wagemans, Nino Grdzelishvili, Nata Bakuradze, Gulnara Natroshvili, Nino Macharashvili, Anahit Sedrakyan, Karine Arakelova, Zhanna Ktsoyan, Magdalina Zakharyan, Zaruhi Gevorgyan, Armine Mnatsakanyan, Farida Tishkova, Cédric Lood, Dieter Vandenheuvel, Rob Lavigne, Jean-Paul Pirnay, Daniel De Vos, Nina Chanishvili, Maia Merabishvili

**Affiliations:** 1 Research & Development Department, George Eliava Institute of Bacteriophage, Microbiology and Virology, Tbilisi 0160, Georgia; elene.kakabadze@pha.ge (E.K.); n.grdzelishvili@pha.ge (N.G.); nata.bakuradze@pha.ge (N.B.); gulikonatroshvili@yahoo.com (G.N.); nina.chanishvili@pha.ge (N.C.); maya.merabishvili@gmail.com (M.M.); 2 Faculty of Exact and Natural Sciences, Ivane Javakhishvili Tbilisi State University, Tbilisi 0179, Georgia; 3 Laboratory of Gene Technology, Department of Biosystems, KU Leuven, 3001 Heverlee, Belgium; jeroen.wagemans@kuleuven.be (J.W.); cedric.lood@kuleuven.be (C.L.); rob.lavigne@kuleuven.be (R.L.); 4 Department of Natural Sciences and Medicine, Ilia State University, Tbilisi 0162, Georgia; 5 Bacteriology Laboratory, Infectious Diseases and AIDS Center, Tbilisi 0160, Georgia; ninomacharashvili459@yahoo.com; 6 Laboratory of Molecular Genetics, Institute of Molecular Biology, National Academy of Sciences of the Republic of Armenia, Yerevan 0014, Armenia; sedanahit@gmail.com (A.S.); karinaraqel@gmail.com (K.A.); ktsoyan@yahoo.com (Z.K.); linazakharyan@gmail.com (M.Z.); 7 Department of Clinical Laboratory Diagnostics, Yerevan State Medical University after Mkhitar Heratsi, Yerevan 0025, Armenia; zaragevorgyan@yahoo.de; 8 Microbiological Laboratory, Nork Infectious Clinical Hospital, Ministry of Health of the Republic of Armenia, Yerevan 0047, Armenia; mnatsakanyan@yahoo.com; 9 Virology Laboratory, Tajik Research Institute of Preventive Medicine, 734025 Dushanbe, Tajikistan; ftishkova@gmail.com; 10 Laboratory of Computational Systems Biology, Department of Microbial and Molecular Systems, KU Leuven, 3000 Leuven, Belgium; 11 Research Group Environmental Ecology and Applied Microbiology, Department of Bioscience Engineering, University of Antwerp, 2020 Antwerp, Belgium; vandenheuveldieter@gmail.com; 12 Laboratory for Molecular and Cellular Technology, Queen Astrid Military Hospital, 1120 Brussels, Belgium; jean-paul.pirnay@mil.be (J.-P.P.); DanielMarie.DeVos@mil.be (D.D.V.)

**Keywords:** *Salmonella*, foodborne pathogens, Armenia, Georgia, bacteriophages, phage therapy, antibiotic resistance, genotyping, genome sequencing, clinical isolates

## Abstract

Non-typhoidal *Salmonella* present a major threat to animal and human health as food-borne infectious agents. We characterized 91 bacterial isolates from Armenia and Georgia in detail, using a suite of assays including conventional microbiological methods, determining antimicrobial susceptibility profiles, matrix assisted laser desorption/ionization-time of flight (MALDI-TOF) mass spectrometry, serotyping (using the White-Kauffmann-Le Minor scheme) and genotyping (repetitive element sequence-based PCR (rep-PCR)). No less than 61.5% of the isolates were shown to be multidrug-resistant. A new antimicrobial treatment strategy is urgently needed. Phage therapy, the therapeutic use of (bacterio-) phages, the bacterial viruses, to treat bacterial infections, is increasingly put forward as an additional tool for combatting antibiotic resistant infections. Therefore, we used this representative set of well-characterized *Salmonella* isolates to analyze the therapeutic potential of eleven single phages and selected phage cocktails from the bacteriophage collection of the Eliava Institute (Georgia). All isolates were shown to be susceptible to at least one of the tested phage clones or their combinations. In addition, genome sequencing of these phages revealed them as members of existing phage genera (*Felixounavirus*, *Seunavirus*, *Viunavirus* and *Tequintavirus*) and did not show genome-based counter indications towards their applicability against non-typhoidal *Salmonella* in a phage therapy or in an agro-food setting.

## 1. Introduction

Food and water-borne diseases represent a growing public health problem worldwide, in both animals and humans. An increasing number of people are at risk of foodborne bacterial infections, often causing severe or even fatal diarrheal diseases, with 550 million people getting ill annually, including 220 million children under the age of five [1]. *Salmonella* is one of the main causative agents of food-borne infections. This ubiquitous and increasingly antibiotic-resistant bacterium [2] can survive several weeks in dry environments and several months in water. While a typical *Salmonella* infection can be resolved without medical treatment, severe cases can have a lethal outcome in the absence of adequate antibiotic treatment.

Drug resistance in non-typhoidal *Salmonella* has been on the rise since 1996 [3]. In 2017, the World Health Organization (WHO) included fluoroquinolone-resistant *Salmonella* spp. in its high-priority pathogens list to guide research and development of new antibiotics [4]. Injudicious use of antimicrobials in veterinary medicine and agriculture has led to multidrug resistance in zoonotic *Salmonella*. Infections caused by resistant strains were found to be more severe, with lower treatment efficacy and higher hospitalization rates [3]. In the worst cases, bacteria spread from the intestines to the bloodstream, causing life-threatening *Salmonella* bacteremia. Certain serotypes are more prone to cause such invasive infections. The United States Centre for Disease Control and Prevention (CDC) analyzed blood and stool *Salmonella* isolates obtained through their surveillance systems in the period 2004–2012 [5] and estimated the overall incidence of resistant *Salmonella* infections as roughly 2 for 100,000 persons per year, with the majority (73%) of the clinically important antimicrobial resistance (AMR) linked to four major *Salmonella* serotypes: Enteritidis, Newport, Typhimurium, and Heidelberg.

In February 2018, sixteen people were diagnosed with severe *Salmonella* food poisoning in Tbilisi, Georgia [6]. This outbreak was associated with chicken burgers sold in a supermarket of a well-known multinational retailer. The Georgian CDC identified the patients’ isolates as *Salmonella enterica* subsp. *enterica* serovar Agona (O:4,12; H1:f,g,s; H2:1,2), according to the Kauffmann-White classification. All isolates showed resistance to ampicillin, two isolates also showed intermediate resistance to nalidixic acid, and one isolate showed a multidrug-resistance phenotype, exhibiting resistance against ampicillin, tetracycline, and nalidixic acid, and intermediate resistance to ciprofloxacin and azithromycin. This example again illustrates that non-typhoidal *Salmonella enterica* is a leading cause of food poisoning in both developed and developing countries and that the emergence of multidrug resistant (MDR) strains represents an additional threat to public health. In the present study, we characterized 91 clinical *Salmonella* isolates, comprising determination of genotype distribution and antibiotic and phage susceptibility profiles. Almost two thirds of the isolates (61.5%, 56/91) were shown to be MDR with 18 isolates (19.7%) showing resistance to third generation cephalosporins and fluoroquinolones.

Therefore alternative means of treatment and prevention of salmonellosis are urgently needed.

Bacteriophages (phages) are considered as additional or complementary tools in the fight against MDR bacteria. Personalized Phage Therapy (PT) has been practiced in Georgia, Russia and Poland for nearly a century [7]. A number of phage preparations (such as Pyo and Intesti Bacteriophage, Eliava Biopreparation, Tbilisi, Georgia), active against various bacterial infections, are available as an over the counter medicine in Georgia. Moreover, the potential of phages to control bacterial pathogens in the agro-food industry has led to the development and marketing of a number of phage products in the United States [8]. Several phage preparations have now been approved as biocontrol agents of food pathogens [9].

Because phages are highly specific and adaptive antimicrobial agents, the investigation and monitoring of target infections, and the creation and maintenance of bacterial collections of well-characterized and epidemiologically relevant clinical isolates, are crucial in generating potent phage preparations.

## 2. Materials and Methods

### 2.1. Initial Isolation and Identification of Salmonella Isolates

One hundred and sixteen bacterial isolates from fecal samples of patients with suspected salmonellosis were obtained from the “Nork” Republican Infectious Clinical Hospital (Yerevan, Armenia; *n* = 77), the Infectious Diseases and AIDS Center (Tbilisi, Georgia; *n* = 25), and the Municipal Infectious Diseases Hospital (Dushanbe, Tajikistan; *n* = 14). The initial diagnoses of salmonellosis were based on clinical presentations: symptom or group of symptoms observed or detected after initial examination or disclosed by a patient to the physician, as well as laboratory analyses. Clinical presentations (anamnesis morbi) consistent with gastroenteritis were diarrhea, fever, nausea, vomiting, and abdominal cramps. For the present investigation, only patients without any therapeutic interference before their hospitalization were selected.

Routine bacteriological analysis of fecal samples was performed. Samples were placed in sterile bottles and processed within one hour after collection. Approximately 0.9 g of fecal material was diluted 1:10 in 0.9% NaCl. Serial 100-fold dilutions of the fecal samples were inoculated on petri plates with Xylose Lysine Deoxycholate (XLD) selective agar (Oxoid Limited, Basingstoke, UK) and incubated for 24 to 48 h at 37 °C. Bacterial identification of the obtained isolates was verified by brightfield microscopy at a ×1000 magnification of Gram-stained bacteria [10]. The nutritional and metabolic capabilities of the isolates were determined to define their genus and species identity. Tests establishing glucose fermentation, urease presence, indole production, H_2_S production, and fermentation of galactitol (dulcitol) were performed according to the UK Standards for Microbiology Investigations [11].

### 2.2. MALDI-TOF MS Identification

Genus level identification of bacterial isolates was performed by matrix assisted laser desorption/ionization-time of flight mass spectrometry (MALDI-TOF MS, MicroFlex™, Bruker Daltonik, MA, USA). Freshly grown bacterial colonies were distributed on a ground steel MALDI target plate using a 1 μL disposable loop. The microbial smears were air-dried and overlaid with 1 μL Bruker IVD Matrix HCCA and further air-dried for 5 min at room temperature. The Bruker MicroFlex instrument was operated using FlexControl 3.0 software (Bruker Daltonik, MA, USA). External calibration of the instrument was performed using the Bacterial Test Standard (BTS, Bruker Daltonik, MA, USA) [12].

### 2.3. Serological Characterization of Salmonella Isolates

Serotyping of isolates was performed in accordance with the White-Kauffmann-Le Minor scheme [13], using polyvalent antisera for flagellar (H) and lipopolysaccharide (O) antigens.

### 2.4. Molecular Typing of Salmonella Isolates

Molecular typing of *Salmonella* isolates was performed by a semi-automated repetitive element sequence-based PCR (rep-PCR) system (DiversiLab^®^ System, bioMérieux, Marcy l’Étoile, France). DNA was extracted using the UltraClean™ Microbial DNA Isolation Kit (MO BIO Laboratories Inc., Solana Beach, CA, USA) according to the manufacturer’s instructions. Rep-PCR was performed using a PTC 200 thermocycler (Applied Biosystems, Nieuwerkerk a/d Ijssel, The Netherlands) and the *Salmonella* fingerprinting kit (Bacterial Barcodes, bioMérieux, Athens, GA, USA). The reaction mixture (total volume 25 μL) consisted of 18 μL rep-PCR MM1, 2.5 μL of Gene Amp PCR buffer 10×, 2 μL of primer Mix, 0.5 μL of AmpliTaq DNA polymerase (Applied Biosystems, Nieuwerkerk a/d Ijssel, The Netherlands) and 2 μL of genomic DNA. Thermal conditions: an initial denaturation step at 94 °C for 2 min, 35 cycles including denaturation at 94 °C for 30 s, annealing at 50 °C for 30 s and extension at 70 °C for 90 s, followed by a final extension step at 70 °C for 3 min. The amplified fragments were separated by electrophoresis using a microfluidic lab-chip. Electropherograms were automatically analyzed using DiversiLab software (version 3.4) (bioMérieux, Brussels, Belgium). All fingerprint patterns were normalized; Pearson correlation (PC) was used to calculate the distance matrices among all samples. Relying on unweighted pair group method with arithmetic mean (UPGMA) clustering and multidimensional scaling, the DiversiLab software created a customized report presenting a dendrogram, electropherograms, virtual gel images and scatter plots. Relatedness among isolates was deduced as previously described [14]; isolates showing similarity levels above 95% were considered as linked, while isolates with similarity levels below 95% were considered as distinct [15].

### 2.5. Antimicrobial Susceptibility Profiles of Salmonella Isolates

The antibiotic susceptibility of the bacterial isolates was determined using the Kirby-Bauer disk diffusion method [16]. The following antibiotic disks (Liofilchem, Roseto degli Abruzzi, Italy) were used: ampicillin (AMP, 10 µg), amoxicillin + clavulanic acid (AUG, 20 µg/10 µg), azithromycin (AZI, 15 µg), ceftriaxone (CRO, 30 µg), chloramphenicol (C, 30 µg), ciprofloxacin (CIP, 5 µg), nalidixic acid (NAL, 30 µg), streptomycin (SM, 10 µg), tetracycline (TE, 30 µg), trimethoprim-sulfamethoxazole (T/S, 1.25 µg/23.75 µg), and sulfamethoxazole (S3, 300 µg). Susceptibility testing results were interpreted based on the Clinical and Laboratory Standards Institute (CLSI) criteria [17].

### 2.6. Bacteriophages and the Propagation Bacterial Strains Used in the Study

**Figure 1:**
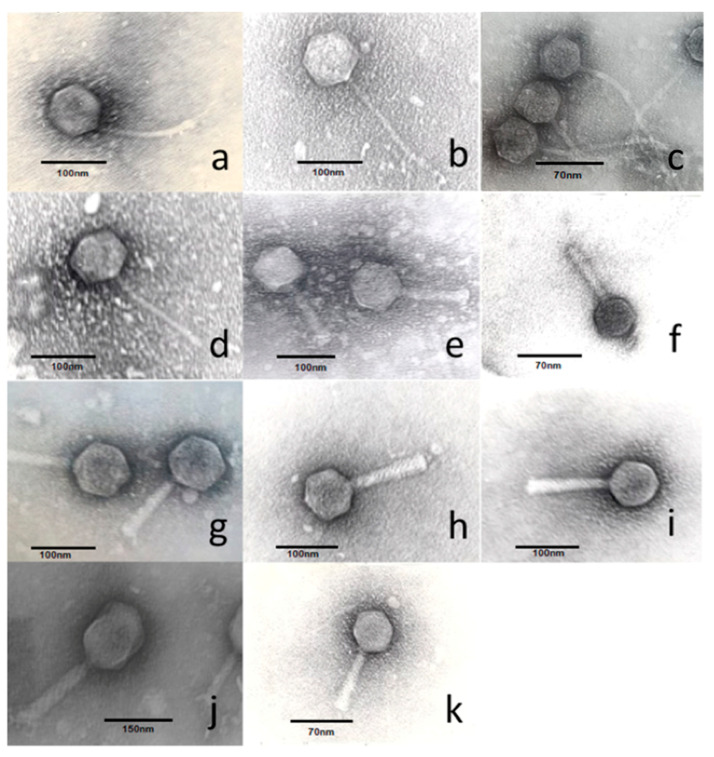
Transmission electron micrographs of bacteriophages used in this study. (**a**) GEC_vB_N3; (**b**) GEC_vB_N5; (**c**) GEC_vB_N7; (**d**) GEC_vB_N8; (**e**) GEC_vB_N6; (**f**) GEC_vB_NS7; (**g**) GEC_vB_MG; (**h**) GEC_vB_BS; (**i**) GEC_vB_B1; (**j**) GEC_vB_GOT; (**k**) GEC_vB_B3.

Eleven *Salmonella* specific single phage clones (GEC_vB_B1, GEC_vB_GOT, GEC_vB_N6, GEC_vB_N7, GEC_vB_N5, GEC_vB_N3, GEC_vB_NS7, GEC_vB_B3, GEC_vB_BS, GEC_vB_MG, GEC_vB_N8) (Table 1 and Figure 1) and three *Salmonella* specific phage cocktails, that were designed using different combinations of three clones selected from the set of eleven phage clones (BTR1, BTR2, BTR3) (Table 2), were screened for activity against the above-mentioned collection of *Salmonella* isolates. These phages were isolated from different environmental sources during the period 2013–2015 (Table 1). Three different bacterial strains (Table 3) were used to propagate the eleven individual phages.

### 2.7. Phage Isolation

Isolation of *Salmonella* specific phages was performed using the bacterial strain enrichment method [18]. Ten ml of 10× concentrated lysogeny broth (LB, Oxoid Limited, Basingstoke, UK) was pipetted into a 125 mL Erlenmeyer flask, 90 mL of the water/milk sample was added and the mixture was inoculated with 1 mL of overnight culture of host bacteria (Table 3). The flask was incubated for 18 h at 37 °C. Then the mixture was centrifuged at 6000× *g* for 30 min at 4 °C and supernatant was filtered through 0.45 or 0.22 µm filters and tested for the presence of phages by a spot test on bacterial streaks [19]. Overnight host bacterial cultures were diluted in the sterile LB to a final concentration of 10^7^ colony forming units (cfu)/mL and streaks were made on 2% LB agar plates using a 10 µL loopful of each strain, and air-dried for 10–15 min. Ten µL of each filtered enrichment sample was applied on each streak. The plates were incubated at 37 °C for 18 h and phage presence was assessed based on visualization of clear spots on the bacterial growth.

### 2.8. Preparation of High-Titer Phage Stocks

To prepare high-titer phage stocks, 0.1 mL containing 10^6^ plaque forming units (pfu) of phages was mixed with 0.1 mL of 10^7^ cfu of host bacteria. The mixture was incubated at room temperature for 10 min and 3–5 mL of 0.7% LB agar at 45 °C was added. The mixture was immediately poured into plates containing 30 mL of solid 2% LB agar. Plates were incubated for 18 to 24 h at 37 °C. When semi-confluent lysis occurred, the top (soft) agar was gently scraped off and collected into a sterile centrifuge tube using a sterile bent Pasteur pipette. The tube was centrifuged at 6000× *g* for 30 min at 4 °C. The supernatant was filtered through 0.45 µm or 0.22 µm filters. The obtained phage stocks were highly concentrated (10^10^ to 10^11^ pfu/mL)

### 2.9. Bacteriophage Susceptibility Test

Assessment of phage activity against different bacterial strains was performed using the parallel streaks method [19,20] with minor modifications. Briefly, overnight bacterial cultures and phage stocks were diluted in the sterile LB to a final concentration of 10^7^ cfu/mL and 10^6^ pfu/mL respectively. Bacterial streaks were made on 2% LB agar plates using a 10 µL loopful of each bacterial isolate and air-dried for 10–15 min. Five µL of each phage clone and cocktail was applied on each streak. The plates were incubated at 37 °C for 18 h and results were recorded. Phage activity was assessed based on visualization. Confluent lysis (CL), semi-confluent lysis (SCL), opaque lysis (OL), countable number of phage plaques on the phage application spots (“taches vierges”, TV) was considered as positive result. Uninterrupted bacterial growth on the spot was recorded as resistant (R). The probability of false-positive phage infection due to lysate impurities (e.g., bacterial toxins) or “lysis from without” was reduced by using defined media and phage production hosts known not to contain bacterial growth suppressing agents in their lysates. In addition, low phage concentration (10^6^ pfu/mL), obtained by dilution of highly concentrated phage stocks (see Section 2.8)—thus strongly diluting potential impurities—were tested. Finally, the spot test often resulted in a countable amount of single phage plaques (TV in Table S1).

### 2.10. Sequencing and Analysis of Phage Genomes

Phage DNA was extracted from a high-titer phage stock by a phenol/chloroform extraction, according to Sambrook and Russell [21]. The phage genomes were subsequently sequenced using an in-house MiniSeq Illumina NGS platform (Illumina, San Diego, CA, USA). The Nextera Flex DNA Library Kit (Illumina) was used for the library prep of the DNA and the concentration was determined with a Qubit fluorometer (Thermo Fisher Scientific, Waltham, MA, USA). After sequencing, trimming and genome assembly was done using the PATRIC (v 3.6.6) server [22]. Using MEGA X [23], phage genomes were aligned to the closest type species as identified by BLASTn [24]. Next, they were annotated using RASTtk [25] and manually curated by BLASTp. Finally, the phage genomes were visualized using EasyFig [26] and SNP variants were called using iVar [27].

## 3. Results and Discussion

### 3.1. Isolation, Identification and Characterization of Salmonella Isolates

A total of 116 bacterial isolates were collected from non-antibiotic-treated patients with presumed salmonellosis in three different countries (Section 2.1). Initially, all 116 isolates were identified as *Salmonella* spp., based on conventional microbiological and biochemical methods. Further MALDI-TOF MS analysis provided a more reliable genus level identification. Ninety-one isolates were confirmed to be *Salmonella* spp., while 25 isolates (14 from Tajikistan, five from Georgia and six from Armenia) appeared to be non-*Salmonella* isolates and were excluded from further analysis. Identification on the species level could not be done by MALDI-TOF and serological testing identified at least 86 of the 91 isolates as *S. enterica* subsp. *enterica*.

More specifically, serotyping based on the White-Kauffmann-Le Minor scheme showed dominance of the Typhimurium serotype among clinical isolates from Georgia and Armenia (no *Salmonella* isolates from Tajikistan could be retained for further analysis). A total of 54 isolates were identified as *S. enterica* subsp. *enterica* serovar Typhimurium, among which ten originated from Georgia and 44 from Armenia. The remaining 10 Georgian and 22 Armenian isolates belonged to the *S*. Enteritidis serotype. For five Armenian isolates, serotypes could not be defined and they were assigned as *Salmonella* spp. We observed an even distribution of two serotypes for Georgian clinical isolates and an increased prevalence of *S*. Typhimurium in isolates from Armenia.

**Figure 2:**
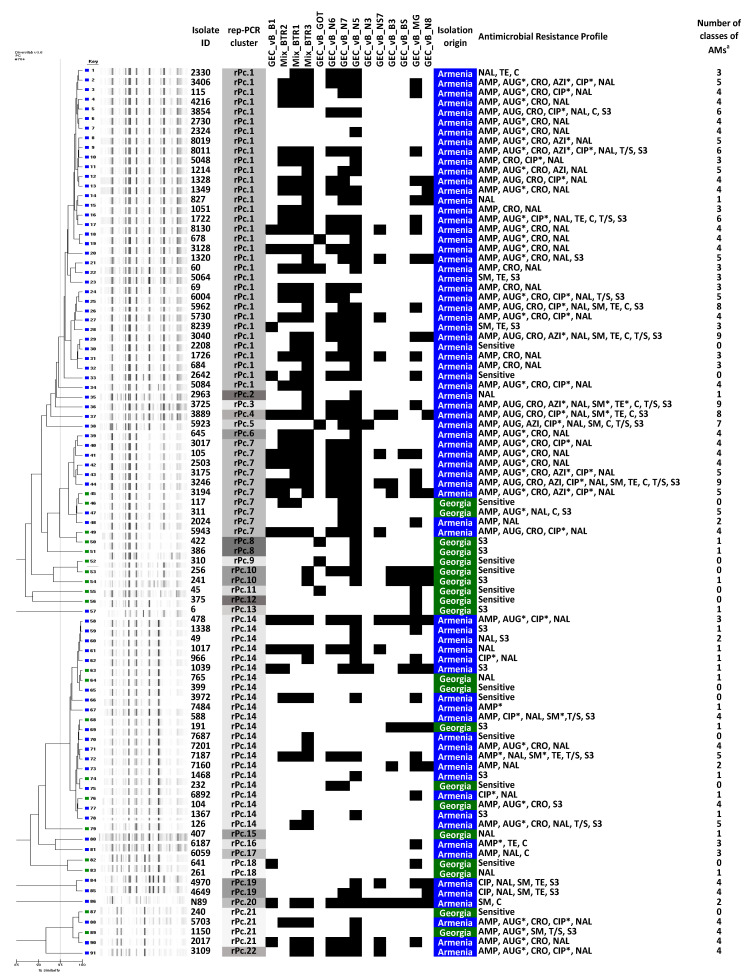
Unweighted pair group method with arithmetic mean (UPGMA) dendrogram of rep-PCR fingerprinting profiles, with corresponding phage typing and AM profiles. The report produced by the DiversiLab software truncated the dendrogram to only show relationships with similarities above 85%. ^a^ Number of classes of AMs to which the isolate showed resistant or intermediate phenotype; * Intermediate susceptibility to AMs. Black nods represent resistance to given phages. Rep PCR clusters are color coded for representation. AM, antimicrobial; AMP, ampicillin; AUG, amoxicillin + clavulanic acid; AZI, azithromycin; C, chloramphenicol; CIP, ciprofloxacin; CRO, ceftriaxone; NAL, nalidixic acid; SM, streptomycin; TE, tetracycline; T/S, trimethoprim-sulfamethoxazole; S3, sulfonamides.

Rep-PCR analysis of the 91 confirmed *Salmonella* isolates revealed 22 distinct genotypic clusters. Three clusters were mixed, harboring both Georgian and Armenian isolates. Cluster rPc.7 consisted of two isolates of Georgian and eight isolates of Armenian origin. Cluster rPc.21 contained two Georgian and two Armenian isolates and cluster rPc.14 contained five Georgian and 17 Armenian isolates (Figure 2). Twenty Georgian isolates clustered in 11 rep-PCR genotypes, of which five were represented by a single strain, and the 71 isolates from Armenia clustered in 14 rep-PCR genotypes, of which nine were represented by a single strain (Figure 2). There was no correlation between rep-PCR cluster compositions and geographical origin or isolation time.

### 3.2. Antimicrobial Susceptibility

**Figure 3:**
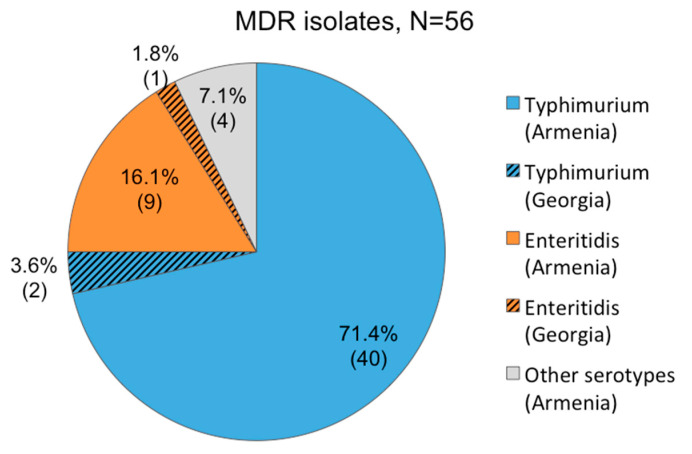
Serotype distribution in multidrug resistant (MDR) *Salmonella enterica* isolates isolated from patients with salmonellosis in Armenia (53 isolates) and Georgia (three isolates).

Antimicrobial susceptibility testing was performed on the 91 MALDI-TOF confirmed *Salmonella* isolates. Strains were considered as MDR when they showed resistance to representatives of at least three antibiotic classes [28]. Distribution of MDR isolates and their antimicrobial resistance profiles are presented in Figure 3 and Table S1. Only three isolates (15%, 3/20) from Georgia exhibited MDR profiles, whereas 53 isolates (74.6%, 53/71) from Armenia were shown to be MDR. The highest levels of resistance among the MDR isolates were observed for nalidixic acid (91.07%, 51/56), the first of the synthetic quinolones, for ampicillin (91.1%, 51/56), for the third-generation cephalosporin ceftriaxone (75%, 42/56), for amoxicillin + clavulanic acid (73.2%, 41/56), for the fluoroquinolone ciprofloxacin (42.9%, 24/56), and for sulfonamide (37.5%, 21/56). An alarming number of isolates showed resistance against antibiotics that are most commonly used to treat *Salmonella* infections, such as third-generation cephalosporins (42 isolates) and fluoroquinolones (24 isolates). Eighteen of the MDR isolates (32.14%, 18/56) showed simultaneous resistance to these classes of antimicrobials. The broadest resistance spectrum was observed in three *S*. Typhimurium isolates from Armenia, which showed resistance against nine antibiotic classes. The most effective antibiotics, at least in vitro, were azithromycin and trimethoprim-sulfamethoxazole with 89.0% (81/91) of isolates showing susceptibility. The least effective was nalidixic acid with only 31.9% (29/91) of isolates exhibiting susceptibility to this antimicrobial agent. Only nine isolates from Georgia and four isolates from Armenia were shown to be sensitive to all antibiotics tested in this study.

### 3.3. Susceptibility of Salmonella Isolates to Different Phages

**Figure 4:**
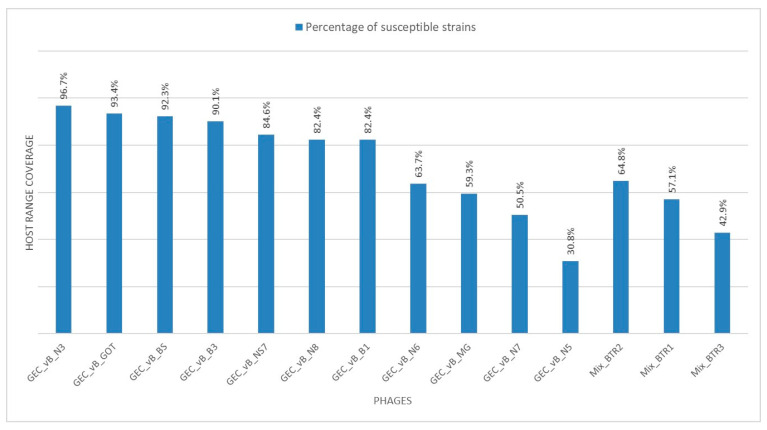
Host range coverages of 11 phage clones and 3 phage cocktails to 91 *Salmonella* isolates from the Caucasus.

All 91 confirmed *Salmonella* isolates were tested for susceptibility to 11 single phage clones and three phage cocktails (Table 1 and Table 2). Of these 11 phage clones, eight were previously shown to have a broad host range against 226 *Salmonella* isolates of veterinary and human origin [29]. Three phages, GEC_vB_GOT, GEC_vB_N6, GEC_vB_N7, have not been reported before. In the present study, all 91 tested *Salmonella* isolates were found to be susceptible to at least one phage. None of the tested strains showed total phage resistance. Figure 4 and Table S1 show the host range coverage of the used phages.

The broadest host range (more than 95% of isolates lysed) was observed for siphovirus GEC_vB_N3. Interestingly, four of the eleven phages screened in this study, GEC_vB_BS, GEC_vB_B1, GEC_vB_B3 and GEC_vB_MG, showed activity against different *Shigella* spp. and *Escherichia coli* isolates, suggesting polyvalency consistent with related species [30].

**Figure 5:**
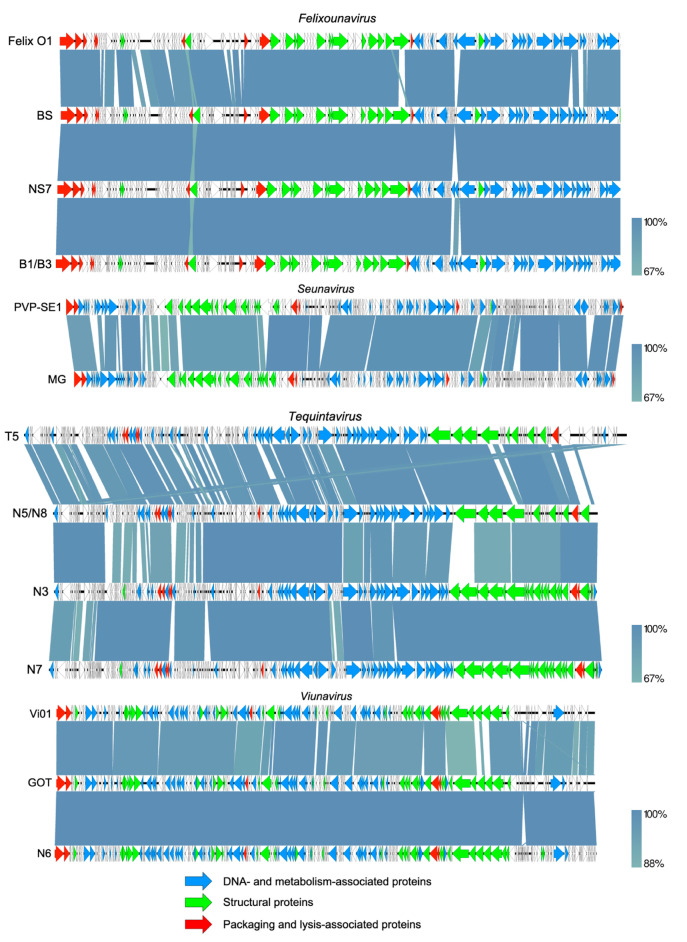
Genome maps of the sequenced *Salmonella* phages and comparison using a BLASTn analysis. The phages can be classified in four different genera: *Felixounavirus* (GEC_vB_BS, GEC_vB_NS7, GEC_vB_B1 and GEC_vB_B3), *Seunavirus* (GEC_vB_MG), *Tequintavirus* (GEC_vB_N5, GEC_vB_N8, GEC_vB_N3 and GEC_vB_N7) and *Viunavirus* (GEC_vB_GOT and GEC_vB_N6). In red, genes encoding packaging and lysis-associated proteins are displayed, in green structural proteins and in blue DNA- and metabolism-associated proteins (adapted EasyFig). Each white or colored arrow represents a predicted open reading frame. Members of the *Tequintavirus* contain direct terminal repeats (DTR) of approximately 10 kb for their packaging strategy, which can be observed by the homology of the last part of the T5 genome to the start of the N5/N8 genomes. For visibility reasons, these redundant DTR regions were deleted from the Genbank files and the figure.

All phages were sequenced individually and their genomes showed high similarities to known phage sequences (Table 1). Phage genome fold coverages ranged from 112X to 364X and all assemblies resulted in circular contigs. The annotated sequences were deposited to NCBI and are available via Genbank accession numbers MW006474 through MW006482. They can be classified in four different genera: *Felixounavirus* (GEC_vB_BS, GEC_vB_NS7, GEC_vB_B1 and GEC_vB_B3), *Seunavirus* (GEC_vB_MG), *Viunavirus* (GEC_vB_GOT and GEC_vB_N6) and *Tequintavirus* (GEC_vB_N5, GEC_vB_N8, GEC_vB_N3 and GEC_vB_N7). The phage genomes were therefore aligned to the type species, representing each genus, and annotated (Figure 5). None of the phages encode genes associated with known lysogeny or virulence and antibiotic resistance determinants. Two morphological types are represented in the analyzed phage set: *Myoviridae* (*n* = 7) and *Siphoviridae* (*n* = 4) (Table 1).

The four phages from the *Felixounavirus*, GEC_vB_BS, GEC_vB_NS7, GEC_vB_B1 and GEC_vB_B3 showed high similarity to the myo-virus Mushroom isolated from the Intestiphage preparation produced by the Eliava Institute [31]. These phages exhibited a broad host range, showing activity against 82%–92% of all tested isolates. Phage GEC_vB_B1 and GEC_vB_B3 are very closely related. When comparing the latter to B1, a heterogenous population can be observed, only displaying some SNPs at a relatively low frequency, which might explain their differences in host range (Table S2). Representatives of the Felix O1-like viruses are well-known, strictly virulent phages active against various enterobacteria, including *E. coli* and *Salmonella* spp. Three out of four phages representing the *Felixounavirus* genus (excluding GEC_vB_NS7), also showed multi species activity. One of the characteristics of the phages from this genus, is a relatively large number of tRNAs (>20) [32], which is also observed in the newly sequenced phage genomes.

*Seunavirus* GEC_vB_MG exhibited a host range of 59.3 % and contains a genome of 142 kb, which showed similarity to the *Salmonella* phages PVP-SE1, the type species of this genus, and to SSE121. Similar to phage PVP-SE1, phage GE_vB_MG not only infects different serotypes of *Salmonella* [33], but also *E. coli* and *Shigella* strains. Phage SSE121 is also known for its activity against *Salmonella* serotypes of high public health importance, including *S*. Typhimurium, *S*. Enteritidis, *S*. Heidelberg, *S*. Newport, and *S*. Hadar. Because of this ability, phage SSE121 is included in the SalmoFresh™ anti-microbial preparation (Intralytix, Baltimore, MD, USA), used for the biocontrol of *Salmonella* in different types of food products.

Phages GEC_vB_N6 and GEC_vB_GOT, respectively with an intermediate (63.7%) and a broad host range (93.4%), showed similarity to representatives of the genus *Viunavirus*, which includes *Salmonella* phages STML-13-1 (98.54% nucleotide identity), Vi01 (96.18%) and PhiSH19 (91.69%). Phage STML-13-1 is known for its broad host range and it is also included in the above-mentioned SalmoFresh™ preparation. Phages Vi01 and PhiSH19 are interesting because of their tail spike proteins [34]. According to Hooton et al. 2011, the variety in number or structure of tail spike protein modules determines host range specificity in the Vi01-like phage genus. Three tail spike protein genes have also been identified within the genomes of phages GOT and N6. Two of them (GOT_Gp163 or N6_Gp179 and GOT_Gp166 or N6_Gp182) reveal high similarity to Vi01 Tsp1 (84.99 and 86.74% identity, respectively) and the Vi01 hemolysin-type calcium-binding protein (99.53% identity), respectively. The second predicted tail spike protein (GOT_Gp165 and N6_Gp181), on the other hand, is similar to the tail spike protein of phage Marshall (86.10% identity), with beta helix/pectin lyase domains resembling the ones found in the tail spikes of the well-known *Salmonella* phage P22 [35]. No genes associated with either toxicity, lysogenicity or antibiotic resistance were found in any phages of the Vi01 genus [31].

The last four phages, GEC_vB_N5, GEC_vB_N8, GEC_vB_N3 and GEC_vB_N7, all belong to the *Tequintavirus* genus, which includes the well-known *E. coli* lytic phage T5 and a number of *Salmonella* phages, such as SPC35, Stitch, Shivani and others [36]. However, these phages revealed very different host ranges. GEC_vB_N5 showed activity against only 30.8% of the tested isolates, while GEC_vB_N8 lysed up to 82.4% of isolates. Activity of GEC_vB_N7 represents 50.5% while GEC_vB_N3 is the most active phage compared to other phages used in this study, lysing 96.7% of strains. On the genome level, however, phage GEC_vB_N5 and GEC_vB_N8 are very closely related. Similar to B1/B3, a heterogenous population can be observed, only displaying a few SNPs at a relatively low frequency (Table S3).

The phage cocktails screened in this study were designed based on the host range distribution of the individual phages. The four phages used in the cocktails (GEC_vB_MG, GEC_vB_N5, GEC_vB_N6 and GEC_vB_N7) belonged to three different genera and showed relatively narrow host ranges (30.8–63.7%), but at the same time exhibited an activity against the isolates that were resistant to other more active phage clones. Hypothetically, mixtures of three of these phages could have host ranges of above 80% (Table S1). In practice, however, the cocktails showed reduced activity against the tested *Salmonella* isolates compared to some individual phage clone components (Table S1). For instance, cocktails Mix_BTR1 and Mix_BTR3 showed an activity of 57.1% and 42.9%, respectively, while the individual component phage GEC_vB_MG was active against 59.3% of the isolates. This emphasizes the fact that therapeutic phage cocktails are best not designed as a mixture of phages, selected solely on phage type and the sum of their individual host ranges [37,38,39]. In designing complex phage therapeutics, the synergetic and antagonistic activity of the different component phages should also be taken into account.

## 4. Conclusions

Clinical non-typhoidal *Salmonella* isolates obtained from the Caucasian region were characterized using phenotypical and molecular methods. Investigation of antibiotic resistance profiles showed an alarming rate of MDR *Salmonella* isolates, including resistance to the third-generation cephalosporins and fluoroquinolones, which are commonly and widely used in the treatment of severe *Salmonella* infections. Because of the increasing rate of AMR in clinical *Salmonella* isolates, new treatment strategies and methods are urgently needed. The application of phages as an additional tool for the treatment of MDR *Salmonella* infections seems to be plausible. Phages are natural and specific antibacterial agents, which can lyse bacteria irrespective of their AMR status, whilst leaving the commensal microflora unharmed. This is one of the main advantages of phages in comparison to antibiotics. The phages tested in this study showed potential for application in phage therapy against MDR *Salmonella* infections.

## Figures and Tables

**Table 1:** List of bacteriophages used in the study.

Phage Name	Genus (Morphology)	Size of Phage Head/Tail (nm)	Genome Size (kb)	NCBI Closest Match	NCBI Query Coverage (%)	NCBI Percent Identity (%)	Isolation Year	Isolation Source/Place
GEC_vB_N3	*Tequintavirus* (*Siphoviridae*)	68/140	110	*Salmonella* phage 1-29	79	97	2013	River Mtkvari, Tbilisi, Georgia
GEC_vB_N5	*Tequintavirus* (*Siphoviridae*)	90/231	149	*E. coli* phage T5	86	97	2013	River Mtkvari, Tbilisi, Georgia
GEC_vB_N7	*Tequintavirus* (*Siphoviridae*)	87/136	112	*Salmonella* phage 1-29	81	95	2013	River Mtkvari, Tbilisi, Georgia
GEC_vB_N8	*Tequintavirus* (*Siphoviridae*)	77/168	51	*E. coli* phage SPC35	84	92	2013	River Mtkvari, Tbilisi, Georgia
GEC_vB_N6	*Viunavirus* (*Myoviridae*)	104/140	158	*Salmonella* phage STML-13-1	90	98	2013	River Mtkvari, Tbilisi, Georgia
GEC_vB_NS7	*Felixounavirus* (*Myoviridae*)	63/109	55	*Salmonella* phage Mushroom	96	99	2015	Cow raw milk, Tbilisi, Georgia
GEC_vB_MG	*Seunavirus* (*Myoviridae*)	95/104	142	*Salmonella* phage PVP-SE1	89	96	2013	Sewage water, Tbilisi, Georgia
GEC_vB_BS	*Felixounavirus* (*Myoviridae*)	77/118	86	*Salmonella* phage Mushroom	98	98	2013	Black Sea, Batumi, Georgia
GEC_vB_B1	*Felixounavirus* (*Myoviridae*)	81/122	87	*Salmonella* phage Mushroom	96	98	2013	River Mtkvari, Tbilisi, Georgia
GEC_vB_GOT	*Viunavirus* (*Myoviridae*)	90/119	157	*Salmonella* phage STML-13-1	90	99	2013	Sewage water, Tbilisi, Georgia
GEC_vB_B3	*Felixounavirus (Myoviridae)*	72/113	87	*Salmonella* phage Mushroom	96	98	2013	River Mtkvari, Tbilisi, Georgia

**Table 2:** Composition of the phage cocktails.

Name of Cocktail	Name of Phages
Mix_BTR1	GEC_vB_MG, GEC_vB__N7, GEC_vB_N5
Mix_BTR2	GEC_vB_N7, GEC_vB_N5, GEC_vB_N6
Mix_BTR3	GEC_vB_MG, GEC_vB_N7, GEC_vB_N6

**Table 3:** Bacterial strains used for propagation of phages.

Strain ID	Species/Serotype	Isolation Source	Isolation Place	Isolation Year	Propagated Phages
SeE.3	*S. enterica* Enteritidis	Pig feces	Pig farm, South Korea	2011	GEC_vB_N3, GEC_vB_N8, GEC_vB_N5, GEC_vB_MG
SeT.4	*S. enterica* Typhimurium	Pig feces	Pig farm, South Korea	2011	GEC_vB_N7, GEC_vB_GOT, GEC_vB_BS
SeT.6	*S. enterica* Typhimurium	Pig feces	Pig farm, South Korea	2011	GEC_vB_N6, GEC_vB_NS7, GEC_vB_B3, GEC_vB_B1

## Supplementary Materials

The following are available online at https://www.mdpi.com/1999-4915/12/12/1418/s1, Table S1: Distribution of MDR isolates and their antimicrobial resistance profiles, Table S2: SNP variant analysis of GEC_vB_B3 and the closely related GEC_vB_B1 using iVar, Table S3: SNP variant analysis of GEC_vB_N8 and the closely related GEC_vB_N5 using iVar.
