# Supplementary material for: Characterization of Salmonella Isolates from Various Geographical Regions of the Caucasus and Their Susceptibility to Bacteriophages
Source: Viruses. 2020 Dec 10;12(12):1418. doi: 10.3390/v12121418 (PMC7764154; doi:10.3390/v12121418)
Supplement: Supplementary file 1 [file viruses-12-01418-s001.zip › Table S2.pdf]

Table S2. SNP variant analysis of GEC_vB_B3 and the closely related GEC_vB_B1 using iVar. Based on a BAM pileup of the B3 reads on the B1 reference genome, the reads were inspected for SNP variants. At relatively low frequencies, actually relating to a significant amount of phages in the phage preparation, SNPs can be observed in several non-coding and coding regions. Only read variants with a sequencing depth >100, a frequency >5% and a p-value <0.05 (Fisher's exact test) were retained.

Position in genome	Reference nucleotide	Changed nucleotide	Frequency in the reads	Region or coding sequence
**14131**	T	-C	0.0647887	non-coding region
**14134**	G	A	0.0669456	non-coding region
**14136**	G	A	0.0995851	non-coding region
**14138**	T	G	0.0846774	non-coding region
**14143**	T	A	0.143396	non-coding region
**14144**	A	T	0.151515	non-coding region
**14268**	T	A	0.0608696	non-coding region
**18725**	T	C	0.0694981	hypothetical protein gp41
**39212**	A	C	0.0694981	structural protein gp78
**47833**	G	A	0.0686275	baseplate assembly protein gp88
**48840**	C	T	0.0522088	baseplate assembly protein gp89
**49209**	G	A	0.0859729	baseplate assembly protein gp89
**58124**	T	G	0.06	DNA ligase gp103
**58181**	G	T	0.0732984	DNA ligase gp103
**58192**	C	A	0.0652174	DNA ligase gp103
**58199**	G	A	0.0663265	DNA ligase gp103
**58205**	A	G	0.0520833	DNA ligase gp103
**58208**	T	G	0.0603015	DNA ligase gp103
**58223**	G	A	0.0575916	DNA ligase gp103
**61282**	T	A	0.0543478	non-coding region upstream of DNA polymerase
**61291**	C	T	0.0574713	non-coding region upstream of DNA polymerase
**61294**	T	G	0.0638298	non-coding region upstream of DNA polymerase
**61297**	A	T	0.0744681	non-coding region upstream of DNA polymerase
**61299**	A	T	0.0625	non-coding region upstream of DNA polymerase
**61300**	A	T	0.0597826	non-coding region upstream of DNA polymerase
**61302**	C	A	0.0760234	non-coding region upstream of DNA polymerase
**61306**	C	T	0.0778443	non-coding region upstream of DNA polymerase
**61312**	G	C	0.0792683	non-coding region upstream of DNA polymerase
**61318**	G	A	0.0731707	non-coding region upstream of DNA polymerase
**62585**	C	T	0.0648148	DNA polymerase gp114
**62609**	C	T	0.0776256	DNA polymerase gp114
**62618**	T	C	0.0737327	DNA polymerase gp114
**62747**	C	T	0.0740741	DNA polymerase gp114
**62810**	T	C	0.0552486	DNA polymerase gp114
**62831**	T	C	0.0581395	DNA polymerase gp114
**62849**	T	C	0.0520833	DNA polymerase gp114
**62894**	C	T	0.0578035	DNA polymerase gp114
**62993**	G	A	0.0670103	DNA polymerase gp114
**63167**	A	G	0.0761905	DNA polymerase gp114
**63188**	T	C	0.0731707	DNA polymerase gp114
**63227**	C	T	0.057971	DNA polymerase gp114
**63245**	A	G	0.0571429	DNA polymerase gp114
