# Supplementary material for: Characterization of Salmonella Isolates from Various Geographical Regions of the Caucasus and Their Susceptibility to Bacteriophages
Source: Viruses. 2020 Dec 10;12(12):1418. doi: 10.3390/v12121418 (PMC7764154; doi:10.3390/v12121418)
Supplement: Supplementary file 1 [file viruses-12-01418-s001.zip › Table S3.pdf]

Table S3. SNP variant analysis of GEC_vB_N8 and the closely related GEC_vB_N5 using iVar. Based on a BAM pileup of the N8 reads on the N5 reference genome, the reads were inspected for SNP variants. At relatively low frequencies, actually relating to a significant amount of phages in the phage preparation, SNPs can be observed in several non-coding and coding regions. Only read variants with a sequencing depth >100, a frequency >5% and a p-value <0.05 (Fisher's exact test) were retained.

Position in genome	Reference nucleotide	Changed nucleotide	Frequency	Region or coding sequence
**84**	C	G	0.129231	non-coding region
**87**	T	A	0.159884	non-coding region
**89**	T	A	0.0513514	non-coding region
**90**	T	A	0.161383	non-coding region
**92**	A	INS	0.121005	non-coding region
**912**	A	T	0.0696379	non-coding region
**3422**	G	A	0.0840108	non-coding region before A1 protein
**3424**	A	T	0.0909091	non-coding region before A1 protein
**3425**	A	C	0.0909091	non-coding region before A1 protein
**3428**	G	T	0.0939948	non-coding region before A1 protein
**3429**	A	T	0.0923483	non-coding region before A1 protein
**3431**	T	C	0.145714	non-coding region before A1 protein
**4694**	A	T	0.184049	non-coding region before hypothetical protein gp8
**4695**	T	A	0.159884	non-coding region before hypothetical protein gp8
**4696**	C	A	0.19195	non-coding region before hypothetical protein gp8
**4697**	G	A	0.175287	non-coding region before hypothetical protein gp8
**4700**	C	T	0.183381	non-coding region before hypothetical protein gp8
**4717**	C	T	0.0676471	non-coding region before hypothetical protein gp8
**4977**	A	G	0.0805195	hypothetical protein gp10
**4994**	A	G	0.216292	hypothetical protein gp10
**4997**	T	C	0.216667	hypothetical protein gp10
**4998**	T	G	0.203297	hypothetical protein gp10
**4999**	T	A	0.196133	hypothetical protein gp10
**5000**	A	T	0.203343	hypothetical protein gp10
**5765**	A	C	0.0519481	non-coding before hypothetical protein gp14
**5779**	A	C	0.13354	non-coding before hypothetical protein gp14
**5788**	C	T	0.12766	non-coding before hypothetical protein gp14
**5799**	A	T	0.104167	non-coding before hypothetical protein gp14
**5800**	A	T	0.1	non-coding before hypothetical protein gp14
**8261**	A	G	0.0509091	hypothetical protein gp19
**9037**	T	C	0.0532544	hypothetical protein gp23
**16154**	A	INS	0.114428	hypothetical protein gp44
**16157**	G	A	0.166667	hypothetical protein gp44
**16158**	T	C	0.125714	hypothetical protein gp44
**21768**	C	A	0.0547264	non-coding region before holin gp55
**27924**	G	A	0.0526316	non-coding region before gp69
**27929**	G	T	0.0582011	non-coding region before gp70
**38033**	C	A	0.0533333	hypothetical protein gp94
**42183**	A	G	0.0821918	hypothetical protein gp106
**42185**	A	INS	0.0568928	hypothetical protein gp106
**42190**	A	T	0.06469	hypothetical protein gp106
**42191**	C	A	0.0611702	hypothetical protein gp106
**42192**	A	T	0.0520833	hypothetical protein gp106
**42194**	A	T	0.0567568	hypothetical protein gp106
**42194**	A	C	0.0540541	hypothetical protein gp106
**42197**	A	T	0.0668449	hypothetical protein gp106
**42198**	C	T	0.0592992	hypothetical protein gp106
**42202**	A	T	0.0641711	hypothetical protein gp106
**42205**	A	T	0.0518135	hypothetical protein gp106

**42206**	A	G	0.0572917	hypothetical protein gp106
**42210**	G	T	0.0603448	hypothetical protein gp106
**42211**	C	INS	0.0512821	hypothetical protein gp106
**42212**	A	T	0.0589888	hypothetical protein gp106
**42217**	C	T	0.0672515	hypothetical protein gp106
**45435**	T	-C	0.0557692	non-coding region before gp112
**80946**	C	T	0.0506912	non-coding region
**80949**	A	T	0.0544218	non-coding region
**80950**	A	G	0.0560748	non-coding region
**81955**	T	G	0.081448	Long tail fiber gp160
**104674**	G	C	0.0526316	non-coding before portal protein gp184
**109610**	A	C	0.0643777	non-coding before receptor-binding protein gp187
**109615**	G	INS	0.0718954	non-coding before receptor-binding protein gp187
**109617**	A	INS	0.0746753	non-coding before receptor-binding protein gp187
**109623**	G	A	0.0836364	non-coding before receptor-binding protein gp187
**109729**	A	C	0.0514286	non-coding before receptor-binding protein gp187
**109732**	A	C	0.0962099	non-coding before receptor-binding protein gp187
**109733**	A	G	0.0982143	non-coding before receptor-binding protein gp187
**109735**	A	+T	0.0825	non-coding before receptor-binding protein gp187
